# Cerebral nitric oxide and mitochondrial function in patients suffering aneurysmal subarachnoid hemorrhage—a translational approach

**DOI:** 10.1007/s00701-020-04536-x

**Published:** 2020-08-25

**Authors:** Arthur Hosmann, Nadja Milivojev, Sergiu Dumitrescu, Andrea Reinprecht, Adelheid Weidinger, Andrey V. Kozlov

**Affiliations:** 1 Department of Neurosurgery, Medical University of Vienna, Vienna, Austria; 2 Ludwig Boltzmann Institute for Experimental and Clinical Traumatology, AUVA Research Center, Vienna, Austria; 3 Laboratory of Navigational Redox Lipidomics and Department of Human Pathology, IM Sechenov Moscow State Medical University, Moscow, Russian Federation

**Keywords:** Nitric oxide, Microdialysis, Mitochondrial function, Subarachnoid hemorrhage

## Abstract

**Background:**

Cerebral ischemia and neuroinflammation following aneurysmal subarachnoid hemorrhage (SAH) are major contributors to poor neurological outcome. Our study set out to investigate in an exploratory approach the interaction between NO and energy metabolism following SAH as both hypoxia and inflammation are known to affect nitric oxide (NO) metabolism and NO in turn affects mitochondria.

**Methods:**

In seven patients under continuous multimodality neuromonitoring suffering poor-grade aneurysmal SAH, cerebral metabolism and NO levels (determined as a sum of nitrite plus nitrate) were determined in cerebral microdialysate for 14 days following SAH. In additional ex vivo experiments, rat cortex homogenate was subjected to the NO concentrations determined in SAH patients to test whether these NO concentrations impair mitochondrial function (determined by means of high-resolution respirometry).

**Results:**

NO levels showed biphasic kinetics with drastically increased levels during the first 7 days (74.5 ± 29.9 μM) and significantly lower levels thereafter (47.5 ± 18.7 μM; *p* = 0.02). Only during the first 7 days, NO levels showed a strong negative correlation with brain tissue oxygen tension (*r* = − 0.78; *p* < 0.001) and a positive correlation with cerebral lactate (*r* = 0.79; *p* < 0.001), pyruvate (*r* = 0.68; *p* < 0.001), glutamate (*r* = 0.65; *p* < 0.001), as well as the lactate-pyruvate ratio (*r* = 0.48; *p* = 0.01), suggesting mitochondrial dysfunction. Ex vivo experiments confirmed that the increase in NO levels determined in patients during the acute phase is sufficient to impair mitochondrial function (*p* < 0.001). Mitochondrial respiration was inhibited irrespectively of whether glutamate (substrate of complex I) or succinate (substrate of complex II) was used as mitochondrial substrate suggesting the inhibition of mitochondrial complex IV. The latter was confirmed by direct determination of complex IV activity.

**Conclusions:**

Exploratory analysis of our data suggests that during the acute phase of SAH, NO plays a key role in the neuronal damage impairing mitochondrial function and facilitating accumulation of mitochondrial substrate; further studies are required to understand mechanisms underlying this observation.

## Introduction

Early brain injury following aneurysmal subarachnoid hemorrhage (SAH) leads to microthrombosis, disruption of the blood-brain barrier, neuronal and endothelial apoptosis, as well as cytotoxic and vasogenic edema [23, 27]. Subsequently, neuroinflammation caused by neuronal death and cisternal lysis of extravasated erythrocytes triggers delayed onset of pathophysiological processes, including cerebral vasospasm and microcirculatory constriction [7, 8, 31, 33].

It is established that endothelin-1 (ET-1) substantially contributes to vasoconstriction upon SAH [1, 50]. Its action is counteracted by increased production of nitric oxide (NO), a potent vasodilator [9, 26]. As a neurotransmitter gas, NO regulates cerebral blood flow and platelet aggregation, leukocyte migration, and smooth muscle cell proliferation [44]. NO is synthetized in the body by three enzymes: endothelial (eNOS), neuronal (nNOS), and inducible (iNOS) NO synthases. eNOS and nNOS are constitutionally expressed and considered to be physiological regulators, acting as neuro-protectives and in an anti-inflammatory manner [11]. In contrast, iNOS is highly upregulated in the presence of inflammatory stimuli in immune cells and neurons, causing detrimental effects by inducing cerebral vasospasm and microthrombus formation [18, 28, 32, 33, 36, 38]. The activity of iNOS can be elevated due to ischemia-mediated activation of hypoxia-inducible factor (HIF)-1a pathway and inflammatory pathways induced by the extravasated blood [14, 49]. Upregulated iNOS generates excessive amounts of NO, which accounts for a majority of NO-mediated secondary injuries after SAH [14]. It has been shown that the induction of iNOS in endothelial cells followed by ET-1 upregulation leads to delayed-onset vasospasm [28]. Furthermore, iNOS is expressed predominantly in macrophages, generating high concentrations of reactive nitrogen species, which are able to damage endothelial cells and shift vasotropic balance in favor of the endothelium-derived vasoconstrictors [41].

Several studies in humans have already measured NO following SAH within the cerebrospinal fluid (CSF) [25, 43] and cerebral interstitial fluid, i.e., microdialysate, [4, 17, 34, 42], showing significantly elevated NO levels. However, the impact of NO on energy metabolism and its role in neuronal injury following SAH is unknown.

Mitochondrion is one of the key targets for cytotoxic effects of NO. NO interacts with complex I and complex IV of the mitochondrial respiratory chain. This NO action inhibits the flow of electrons through the electron transport chain causing a decrease in ATP synthesis and an enhanced generation of reactive oxygen species [22]. Therefore, we tried to explore in this translational study whether excessive amounts of NO generated upon SAH are sufficient to impair mitochondrial function and subsequently contribute to neuronal injury.

## Materials and Methods

### Population

The study was approved by the ethical committee of the Medical University of Vienna (reference number 1871/2014/Amendment). Multimodality monitoring was performed in all included patients as part of standard care, and NO was determined retrospectively from the microdialysate remnants of routine microdialysis monitoring.

Seven consecutive patients were included, suffering poor-grade aneurysmal SAH, under sedation and mechanical ventilation for maximum cerebroprotection, requiring cerebral multimodality neuromonitoring.

SAH and saccular aneurysm were initially confirmed upon admission by cranial computed tomography (CT) and CT angiography (CTA) followed by digital subtraction angiography (DSA). The aneurysm was treated either by endovascular coiling or surgical clipping within the first 72 h by neurosurgeons cross-experienced in microsurgical and endovascular techniques. External ventricular drainage was placed in all patients for hydrocephalus at admission. Long-term sedoanalgesia was initiated with continuous propofol and remifentanil infusion and was switched to midazolam and sufentanil after 3–5 days. In case of inadequate sedation, ketamine was added. At clinical suspicion for secondary ischemia, CTA and/or DSA were performed to confirm cerebral vasospasm. Diminution of the anterior cerebral artery (ACA) or middle cerebral artery (MCA) ipsilateral to the multimodality probe to diameters smaller than 50% of the physiological lumen at admission were considered as CTA/DSA-vasospasm.

Multimodality monitoring was started at a median interval of 2 days after the first ictus (IQR 2–3.5 days) and performed for a median interval of 18 days (IQR 12.5–19 days). Cerebral metabolism (lactate, pyruvate, glucose, glutamate, glycerol), brain tissue oxygen tension (ptiO_2_), intracranial pressure (ICP), and cerebral perfusion pressure (CPP) were measured bedside. The observation period was divided into the acute phase (day 2–7) and the subacute period (day 8–14), based on the onset of cerebral vasospasm [47].

### Multimodality neuromonitoring

ICP and ptiO_2_ was measured using a NEUROVENT-PTO 2L catheter (Raumedic AG, Helmbrechts, Germany) placed into the brain parenchyma side by side with a microdialysis probe through a two-lumen Bolt (BOLT KIT PTO 2L, Raumedic AG, Helmbrechts, Germany). Probes were placed into the watershed of the anterior and middle cerebral artery (ACA–MCA) ipsilateral to the ruptured aneurysm and/or maximal extension of subarachnoid blood clot. Median depth of the microdialysis probe’s tip from dura was 32 mm (IQR 30–32 mm). Intracranial hypertension was defined as ICP values of > 20 mmHg and brain tissue hypoxia as ptiO_2_ values of < 15 mmHg.

Bedside transcranial Doppler (TCD), examinations measuring mean flow velocities in the middle cerebral artery ipsilateral to the probe were carried out daily. TCD flow velocities > 120 cm/s were considered as TCD-vasospasm. CPP was recorded continuously and averaged for each day.

### Cerebral microdialysis

A microdialysis probe (70 MD Catheter, M Dialysis AB, Stockholm, Sweden) was perfused at a flow rate of 0.3 μL per minute by a microinfusion pump (107 Microdialysis Pump, M Dialysis AB, Stockholm, Sweden) filled with Perfusion Fluid CNS (M Dialysis AB, Stockholm, Sweden). Microdialysis sampling started 3 h after probe insertion to exclude neurochemical changes due to implantation trauma [12]. Samples were collected in Microvials (M Dialysis AB, Stockholm, Sweden), analyzed bedside every hour and stored at − 21 °C thereafter. The microdialysis analyzer (ISCUSflex, M Dialysis AB, Stockholm, Sweden) was used for immediate colorimetric neurochemical analysis of glucose, lactate, pyruvate, glycerol, and glutamate concentrations. For further analysis, microdialysis parameters were averaged for each day. The lactate-pyruvate ratio (LPR) was calculated as an established marker of cellular redox status between aerobic and anaerobic energy conservation.

### Determination of NOx in microdialysate fluid and NO solutions

Concentrations of NOx (NO_2_^−^ + NO_3_^−^ + SNO^−^) in microdialysis samples were determined using chemiluminescence-based assay on Sievers 280i Nitric Oxide Analyzer (General Electrics, Boulder, CO). Prior to analysis, the trap chamber was filled with 1 N NaOH (Sigma-Aldrich, Steinheim am Albuch, Germany) and the reflux chamber with the reduction agent (0.8% vanadium (III) chloride (Sigma-Aldrich) in 1 N HCl (Sigma-Aldrich). The latter was used to reduce all NOx species to nitric oxide. The temperature of the reaction chamber was set to 95 °C. To quantify the NOx levels, sodium nitrite standards were used, prepared as aqueous solution of NaNO_2_ (Sigma-Aldrich) in a concentration range of 10 nM–10 μM.

### Ex vivo analysis of mitochondrial function

Respiratory parameters of mitochondria were monitored using high-resolution respirometry (Oxygraph-2k, Oroboros Instruments, Innsbruck, Austria). Male Sprague-Dawley rats (300–350 g, *n* = 3) were anesthetized with 3% isoflurane (Abbott, Austria). After decapitation, the brain was excised and immediately placed in ice-cold Ringer solution (Fresenius Kabi, Austria). For preparation of rat cortex homogenates, 300 mg of cortex tissue were homogenized (RW 1 basic homogenizer, IKA, USA) with 3 volumes (1:4 wt/vol) of preparation buffer (250 mM saccharose, 10 mM Tris, 0.5 mM EDTA, 0.05% BSA, pH 7.2). For measurement of mitochondrial respiration rate, rat cortex homogenates were incubated in buffer containing 105 mM KCl , 5 mM KH_2_PO_4_, 20 mM Tris-HCl, 0.5 mM EDTA, and 5 mg/mL fatty acid-free bovine serum albumin (pH 7.2, 37 °C). Complex I-linked state 3 respiration was stimulated by addition of 10 mM glutamate and 1 mM adenosine diphosphate (ADP). Complex II-linked state 3 respiration was then stimulated with 10 mM succinate after addition of complex I inhibitor rotenone [1 ng/mL]. Complex IV activity was measured after the addition of ascorbate (200 μM) and tetramethyl-p-phenylenediamine dihydrochloride (500 μM). Sodium azide (200 mM) was used to correct for autooxidation. Myxothiazol (10 μM), a complex III inhibitor, was used to inhibit mitochondrial oxygen consumption. Respiration rates were obtained by calculating the negative time derivative of the measured oxygen concentration.

NO solution was prepared by bubbling NO gas through the buffer solution in the absence of oxygen. The levels of NO in the stock solution were determined by NO analyzer as described above without reductive medium. Nitric oxide solution (0.5 mM) was added at final concentrations of 5 μM, 25 μM, and 50 μM.

### Statistical analysis

Statistical analysis was performed in an explorative/descriptive approach using SPSS® Statistics 22 (IBM Corp., Armonk, NY). Metric data are presented as mean ± standard deviations. Continuous data was averaged for each day following SAH and presented descriptively. Subgroup analysis was performed in an explorative approach. For group comparison of normally distributed metric variables (assessed by the Kolmogorov-Smirnov test), the independent *t* test was used, whereas Mann-Whitney *U* test was used if they were not normally distributed. For correlation, the Pearson correlation coefficient (*r*) analysis was performed. Mitochondrial respiration data were analyzed using GraphPad Prism software (GraphPad Software 5.01, USA) by one-way ANOVA followed by Dunnett’s multiple comparison test (all groups vs. control). Differences were considered to be statistically significant at a two-sided significance level of *α* < 0.05.

## Results

### Population

Patients’ characteristics are shown in Table 1. Mean multimodality neuromonitoring parameters for the observation period between day 2 and day 14 are shown in Table 2. TCD-vasospasm ipsilateral to the multimodality probe was present in five patients. Highest TCD flow velocities were observed on day 10 after SAH (138.2 ± 43.4 cm/s). In total, 10 CTA/DSA examinations were performed at a mean interval of 10.2 ± 2.9 days. CTA/DSA revealed cerebral vasospasm in the ipsilateral ACA/MCA-territory in all patients.

**Table 1:** Patients’ characteristics

Parameter	Median (IQR)
Demographics	
Age (years)	53.0 (50–56)
Sex	
Female	5
Male	2
Hunt & Hess at admission	4 (3–4)
mRS 3 months after SAH	3 (1.5–5)
Ruptured aneurysm side	
Anterior communicating artery	3
Middle cerebral artery	3
Posterior communicating artery	1
Aneurysm treatment	
Clipping	4
Coiling	3
Multimodality neuromonitoring	
Start after SAH (days)	2 (2–3.5)
End after SAH (days)	18 (12.5–19)
Probe side in relation to ruptured aneurysm	
Ipsilateral	6
Contralateral	1
Depth of microdialysis probe tip (from dura; mm)	32 (30–32)

Data are shown as median and interquartile range (IQR) or total counts. ACA, anterior cerebral artery; MCA, middle cerebral artery; mRS, modified Rankin scale; SAH, subarachnoid hemorrhage

**Table 2:** Mean values of all multimodality neuromonitoring parameters within 14 days following SAH

Days after SAH	NOx (μM)	ptiO_2_ (mmHg)	TCD (cm/s)	CPP (mmHg)	ICP (mmHg)	Glucose (mmol/L)	Lactate (mmol/L)	Pyruvate (μmol/L)	Glycerol (μmol/L)	Glutamate (μmol/L)	LPR
2	96.0 ± 1.0	14.4 ± 16.7	66.0 ± 5.7	74.9 ± 6.9	6.8 ± 0.9	1.5 ± 0.5	3.9 ± 0.0	113.0 ± 25.6	109.1 ± 54.4	12.5 ± 0.3	35.2 ± 7.9
3	74.5 ± 0.7	19.7 ± 9.9	87.0 ± 25.3	71.6 ± 6.8	7.5 ± 2.2	1.5 ± 0.2	3.7 ± 0.8	111.0 ± 13.4	115.4 ± 63.0	9.3 ± 3.8	33.7 ± 5.0
4	67.3 ± 0.8	26.9 ± 6.6	71.4 ± 39.7	70.5 ± 8.8	9.6 ± 4.1	1.5 ± 0.3	3.9 ± 1.2	116.8 ± 22.1	100.6 ± 44.7	6.7 ± 9.7	32.9 ± 5.5
5	83.7 ± 0.4	23.1 ± 7.0	84.0 ± 42.3	78.6 ± 13.4	8.4 ± 4.8	1.2 ± 0.4	4.0 ± 1.2	119.2 ± 36.7	75.7 ± 23.8	7.0 ± 13.5	33.7 ± 4.0
6	71.4 ± 0.6	22.4 ± 5.5	88.7 ± 49.9	82.9 ± 14.6	7.4 ± 5.0	1.0 ± 0.4	3.7 ± 1.6	118.5 ± 43.2	59.5 ± 13.2	7.6 ± 16.5	30.9 ± 3.5
7	71.8 ± 1.9	21.9 ± 5.5	114.0 ± 67.0	86.0 ± 15.8	8.1 ± 3.9	1.0 ± 0.5	3.7 ± 1.2	120.7 ± 35.3	75.8 ± 48.3	8.4 ± 18.7	30.5 ± 3.6
8	73.1 ± 0.8	23.0 ± 5.1	131.4 ± 59.4	90.3 ± 22.2	6.3 ± 5.6	0.9 ± 0.4	3.9 ± 1.1	124.7 ± 33.5	68.5 ± 37.2	11.6 ± 28.1	31.2 ± 2.9
9	60.5 ± 1.1	22.8 ± 5.1	131.6 ± 56.9	91.0 ± 18.1	6.0 ± 5.4	0.8 ± 0.2	4.3 ± 1.4	127.3 ± 41.4	71.4 ± 42.2	10.5 ± 24.9	33.7 ± 2.4
10	47.0 ± 1.4	20.7 ± 6.2	138.2 ± 43.4	91.4 ± 14.1	7.1 ± 4.0	0.7 ± 0.2	4.4 ± 1.7	124.5 ± 49.3	53.1 ± 26.1	12.5 ± 30.6	35.6 ± 4.8
11	42.9 ± 1.2	20.1 ± 6.7	119.7 ± 43.0	90.6 ± 14.0	7.6 ± 5.2	0.8 ± 0.2	4.8 ± 2.0	124.6 ± 53.6	39.9 ± 16.0	16.3 ± 40.3	38.9 ± 5.5
12	39.3 ± 0.6	22.8 ± 8.7	123.7 ± 62.6	91.8 ± 11.2	7.2 ± 5.0	0.9 ± 0.2	4.6 ± 1.8	122.8 ± 48.7	39.6 ± 16.2	14.0 ± 33.8	38.6 ± 6.6
13	34.6 ± 0.5	21.9 ± 6.4	126.1 ± 69.4	97.0 ± 11.3	6.9 ± 4.6	0.7 ± 0.2	4.4 ± 2.0	113.9 ± 45.3	44.4 ± 21.2	17.6 ± 43.3	38.4 ± 7.8
14	35.1 ± 0.8	23.1 ± 5.5	118.6 ± 50.7	100.9 ± 15.7	5.4 ± 3.8	0.9 ± 0.3	4.3 ± 2.1	98.5 ± 27.6	47.5 ± 23.9	20.6 ± 42.2	43.0 ± 9.6

CPP, cerebral perfusion pressure; ICP, intracranial pressure; LPR, lactate-pyruvate ratio; NOx, nitric oxide; ptiO2, brain tissue oxygen tension; SAH, subarachnoid hemorrhage; TCD, transcranial Doppler ultrasound flow velocities. Data are shown as mean ± standard deviation.

### Nitric oxide and its relation to cerebral oxygenation and metabolism

Within the first 14 days after SAH, mean NOx concentrations were 61.3 ± 19.8 μM. NOx was significantly higher during cerebral hypoxia (101.5 ± 39.2 μM) in comparison with normoxia (51.8 ± 26.7 μM; *p* < 0.001). Levels of ptiO_2_ were significantly lower at pathological NOx levels > 61 μM (17.8 ± 6.8 mmHg vs. 24.2 ± 5.4 mmHg, *p* < 0.001). Elevated NOx levels were associated with significantly lower TCD flow velocities (90.8 ± 40.2 cm/s vs. 123.1 ± 56.8 cm/s, *p* = 0.01). Furthermore, at elevated NOx, the levels of lactate, pyruvate, and glycerol were increased (Table 3).

**Table 3:** Differences of monitoring parameters during cerebral hypoxia and increased nitric oxide levels

		NOx (μM)	ptiO_2_ (mmHg)	TCD (cm/s)	ICP (mmHg)	Glucose (mmol/L)	Lactate (mmol/L)	Pyruvate (μmol/L)	Glycerol (μmol/L)	Glutamate (μmol/L)	LPR
ptiO_2 _(mmHg)	≤ 15	101.5 ± 39.2	11.4 ± 3.3	75.0 ± 25.5	5.3 ± 1.4	1.0 ± 0.4	5.2 ± 1.0	133.7 ± 33.4	81.5 ± 56.7	11.8 ± 16.1	39.8 ± 5.1
	> 15	51.8 ± 26.7	23.7 ± 5.3	118.0 ± 54.8	7.5 ± 4.6	1.0 ± 0.4	4.0 ± 1.5	117.1 ± 38.3	62.0 ± 34.1	12.1 ± 28.6	34.4 ± 6.0
		***p*** **< 0.001**	***p*** **< 0.001**	***p*** **= 0.03**	*p* = 0.06	*p* = 0.67	***p*** **= 0.003**	*p* = 0.11	*p* = 0.52	*p* = 0.48	***p*** **= 0.004**
NOx (µM)	> 61	96.5 ± 30.0	17.8 ± 6.8	90.8 ± 40.2	6.3 ± 2.0	1.0 ± 0.4	4.9 ± 1.2	138.4 ± 31.1	80.9 ± 45.0	17.0 ± 26.2	35.7 ± 5.5
	≤ 61	39.6 ± 10.7	24.2 ± 5.4	123.1 ± 56.8	7.7 ± 5.1	0.9 ± 0.4	3.8 ± 1.5	109.9 ± 37.6	56.5 ± 31.4	9.7 ± 27.6	34.8 ± 6.5
		***p*** **< 0.001**	***p*** **< 0.001**	***p*** **= 0.01**	*p* = 0.11	*p* = 0.51	***p*** **< 0.001**	***p*** **< 0.001**	***p*** **= 0.01**	*p* = 0.60	*p* = 0.55

ICP, intracranial pressure; LPR, lactate-pyruvate ratio; NOx, nitric oxide; ptiO2, brain tissue oxygen tension; TCD, transcranial Doppler ultrasound flow velocities. Data are shown as mean ± standard deviation. Significant *p* values are shown in bold

### Time course of nitric oxide after subarachnoid hemorrhage

**Fig. 1:**
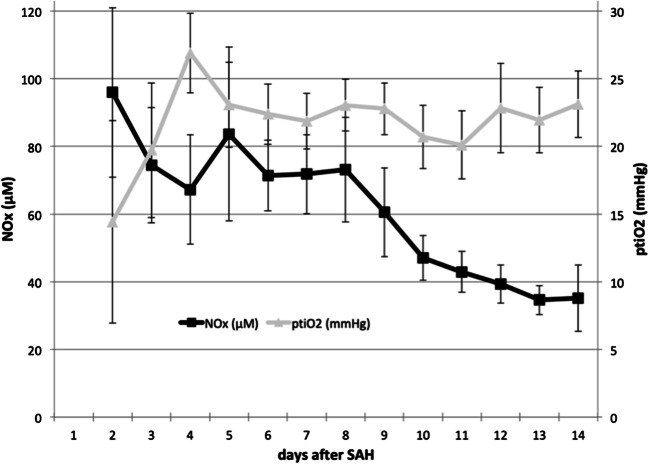
Time course of nitric oxide following subarachnoid hemorrhage. Mean nitric oxide (NOx) and brain tissue oxygen (ptiO_2_) values are displayed within 14 days after subarachnoid hemorrhage (data are shown as mean ± SEM). SAH, subarachnoid hemorrhage

NOx decreased from initial 96.0 ± 56.0 μM to a plateau between day 3 to 8 (73.6 ± 37.5 μM) and declined gradually thereafter to 35.1 ± 21.9 μM on day 14 (Fig. 1). Levels of NOx showed biphasic kinetics being strongly increased during the acute phase (74.5 ± 29.9 μM) and significantly lower during the subacute period (47.5 ± 18.7 μM; *p* = 0.02). LPR showed a reversed pattern with significantly lower values during the first week (31.3 ± 3.8) compared with the second week after SAH (36.6 ± 5.0, *p* = 0.02). However, there was no difference in mean ptiO_2_ values between acute (21.9 ± 5.2 mmHg) and subacute period (22.0 ± 5.6 mmHg, *p* = 0.74).

### Correlation analysis of nitric oxide during the acute and subacute period

Correlations between NOx and metabolic values (i) during the entire observation period as well as during (ii) the acute and (iii) subacute phases are shown in Table 4. Overall, NOx had a strongly negative correlation with brain tissue oxygenation (*r* = − 0.45; *p* < 0.001), TCD flow velocities (*r* = − 0.33; *p* = 0.003), and a positive correlation with cerebral lactate (*r* = 0.31; *p* = 0.01), pyruvate (*r* = 0.39; *p* < 0.001), and glycerol (*r* = 0.32; *p* = 0.01). These correlations were more pronounced during the acute phase, except for TCD and glycerol, which did not show a significant correlation. In addition, LPR (*r* = 0.483; *p* = 0.01) and glutamate (*r* = 0.650; *p* < 0.001) revealed a strong positive correlation with NOx during the acute phase.

**Table 4:** Correlation of NOx with multimodality monitoring parameters within the first 14 days following subarachnoid hemorrhage

NOx	ptiO_2_	TCD	CPP	ICP	Glucose	Lactate	Pyruvate	Glycerol	Glutamate	LPR
Days 1–14	− 0.447	− 0.334	− 0.207	− 0.173	0.172	0.312	0.393	0.319	0.157	− 0.065
	***p*** **< 0.001**	***p*** **= 0.003**	*p* = 0.07	*p* = 0.14	*p* = 0.14	***p*** **= 0.01**	***p*** **< 0.001**	***p*** **= 0.01**	*p* = 0.17	*p* = 0.58
Days 1–7	− 0.776	− 0.210	− 0.134	− 0.408	0.057	0.790	0.684	0.321	0.650	0.483
	***p*** **< 0.001**	*p* = 0.28	*p* = 0.49	***p*** **= 0.03**	*p* = 0.77	***p*** **< 0.001**	***p*** **< 0.001**	*p* = 0.09	***p*** **< 0.001**	***p*** **= 0.01**
Days 8–14	− 0.215	− 0.251	0.028	− 0.174	− 0.178	0.255	0.337	0.019	0.119	− 0.113
	*p* = 0.15	*p* = 0.09	*p* = 0.85	*p* = 0.24	*p* = 0.23	*p* = 0.08	***p*** **= 0.02**	*p* = 0.90	*p* = 0.42	*p* = 0.45

CPP, cerebral perfusion pressure; ICP, intracranial pressure; LPR, lactate-pyruvate ratio; NOx, nitric oxide; ptiO_2_, brain tissue oxygen tension; TCD, transcranial Doppler ultrasound flow velocities. Significant *p* values are shown in bold

### Ex vivo mitochondrial function

**Fig. 2:**
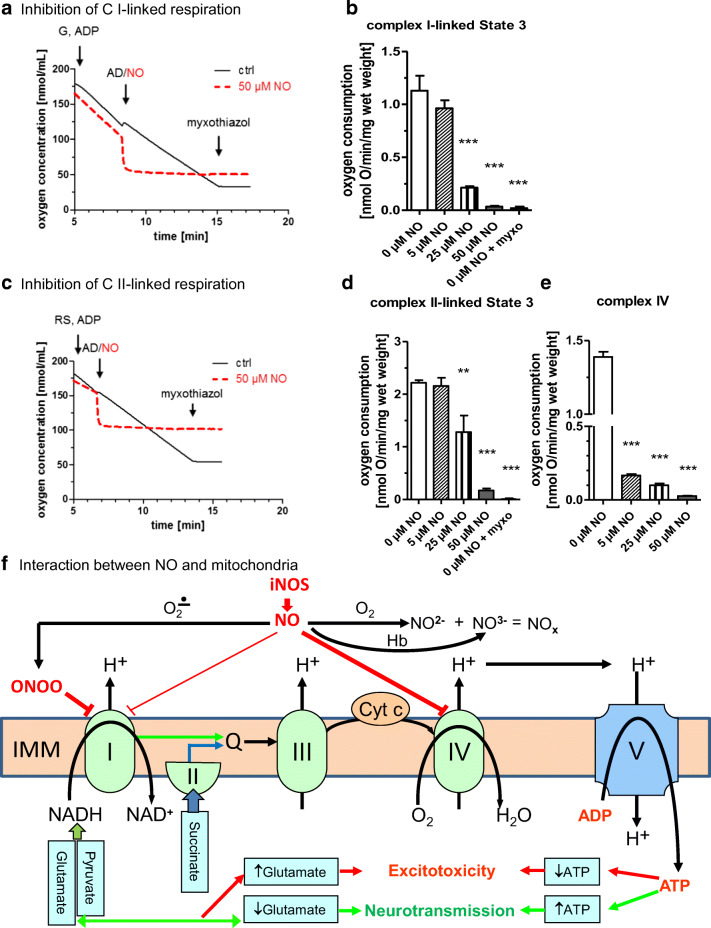
The impact of nitric oxide on mitochondrial respiration. Changes in oxygen concentrations were monitored using the Oroboros Oxygraph-2k (Oroboros Instruments, Austria). Representative traces of changes in oxygen concentration (**a**, **c**) and oxygen consumption graphs (**b**, **d**, **e**) of complex I-linked state 3 respiration (**a**, **b**), complex II-linked state 3 respiration (**c**, **d**), and complex IV (**e**). Rat cortex tissue was homogenized, and mitochondrial state 3 respiration, reflecting adenosine triphosphate (ATP) synthesis, was stimulated by either addition of complex I substrate glutamate (G, 10 mM) and adenosine diphosphate (ADP, 1 mM) (**a**, **b**) or complex II substrate succinate (10 mM) and ADP (1 mM) after addition of complex I inhibitor rotenone (1 ng/mL; RS, rotenone/succinate) (**c**, **d**). Complex IV activity was stimulated by the addition of tetramethyl-p-phenylenediamine dihydrochloride (200 μM, **e**). Nitric oxide (NO) solution was added in final concentrations of 5 μM, 25 μM, and 50 μM (**b**, **d**, **e**). Myxothiazol (myxo, 10 μM) was used to inhibit mitochondrial oxygen consumption (**a–d**). Representative oxygen concentration traces showing that 50 μM NO almost fully inhibited oxygen consumption (dashed line) compared with control (solid line) (**a**, **c**). *n* = 3, mean ± SEM. (**f**) Schematic presentation of the interaction between NO and mitochondria in SAH**.** NO is synthetized by one of the nitric oxide synthase (NOS) family enzymes, predominantly by inducible nitric oxide synthase (iNOS) upon inflammatory response. iNOS is associated with inflammatory reactions rather than with regulation vascular tonus. NO formed may reversibly react with its targets such as mitochondrial complexes or being oxidized by oxygen to nitrite (NO^2−^) or by hemoglobin (Hb) to nitrate (NO^3−^). The sum of NO^2−^plus NO^3−^formed in the tissue is the measure of total NO formed (NOx) determined in this study. Mitochondrial complexes are the key target for excessive NO levels formed by iNOS. Five mitochondrial complexes (I, II, III, IV, V) responsible for adenosine triphosphate (ATP) synthesis are located in the inner mitochondrial membrane (IMM). NO may react with superoxide radical to form peroxynitrite (ONOO), which primarily inhibits complex I, while NO itself predominantly binds to complex IV preventing electron transport to oxygen. Block of mitochondrial respiratory chain will result in decreased uptake of substrates from tricarboxylic acid cycle, such as pyruvate and glutamate and cause a drop in ATP levels. Elevated levels of glutamate and decreased levels of ATP will induce excitotoxicity and neuronal death.

The addition of 5 μM NO showed no effect on mitochondrial complex I (glutamate, Fig. 2b) or complex II (succinate, Fig. 2d)-linked respiration. In contrast, the addition of 25 μM NO caused a substantial decrease in complex I (Fig. 2b, *p* < 0.001) and complex II (Fig. 2d, *p* = 0.01)-linked respiration where the effect was more pronounced when glutamate was used as substrate. An NO concentration of 50 μM nearly fully inhibited mitochondrial respiration irrespectively whether substrate of complex I (glutamate, Fig. 2a, b) or complex II (succinate, Fig. 2c, d) were provided (*p* < 0.001). In NO samples, almost no effect was observed after addition of myxothiazol. In contrast, in control samples, the addition of myxothiazol caused a strong decrease of mitochondrial respiratory activity (Fig. 2b and d). Measurement of complex IV activity showed that the addition of 5 μM, 25 μM, or 50 μM NO caused a strong continuing decrease in the oxygen consumption rate (Fig. 2e, *p* < 0.001).

## Discussion

**Fig. 3:**
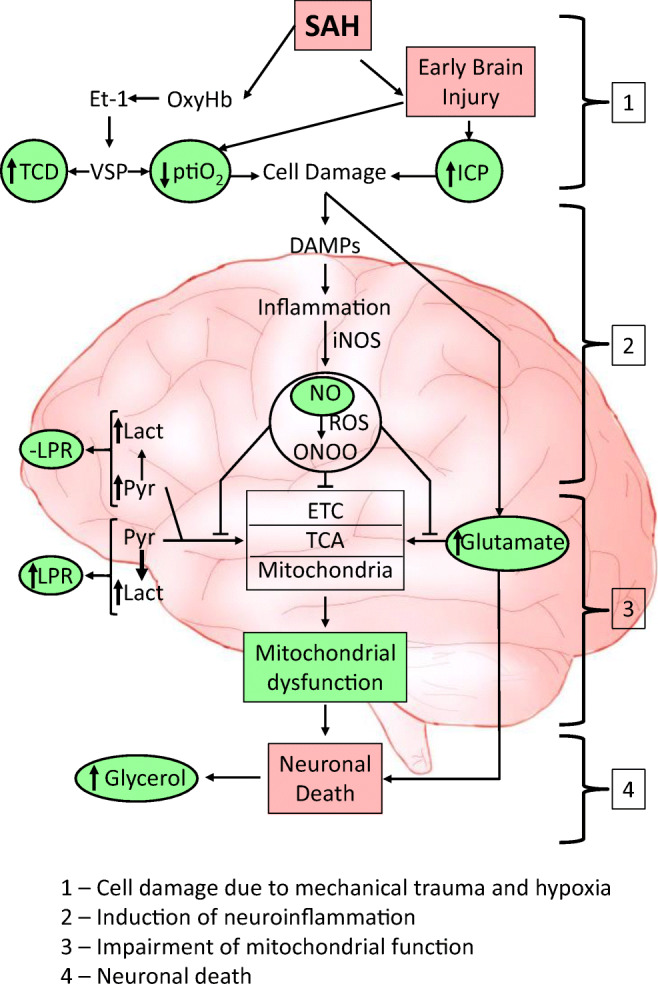
Pathophysiological mechanisms of nitric oxide following subarachnoid hemorrhage. The data obtained in this study are well fitting to the following scheme. Initially subarachnoid hemorrhage (SAH) induces cell damage due to mechanical compression of the brain tissue and induction of ischemia due to the endothelin-1 (ET-1)-induced vasospasm. Three markers (TCD, ptiO_2_, ICP) enabled us to monitor this phase. Green background marks the values determined in this study. During the next phase, damaged cells are supposed to release damage-associated molecular patterns (DAMPs), which activate aseptic inflammation in the brain tissue. In this study, we did not monitor specific markers of inflammation, except nitric oxide (NO). The induction of inflammation, however, was already well established in numerous publications. The most critical event in this scheme is the inhibition of mitochondria by NO (shown in details in Fig. 2), resulting in the disruption of the energy metabolism and accumulation of glutamate. This phase we monitored by measuring LPR and glutamate and the proof of principle the inhibition of mitochondria was confirmed in ex vivo experiment. The last phase, the neuronal damage/death, was monitored by the release of glycerol. Abbreviations: DAMPs, damage-associated molecular patterns, ETC, electron transport chain; ET-1, endothelin-1; ICP, intracranial pressure; iNOS, inducible nitric oxide synthase; Lact, lactate LPR, lactate-pyruvate ratio; NO, nitric oxide; ONOO, peroxynitrite; OxyHb, oxyhemoglobin; pitO_2_, brain tissue oxygen; Pyr, pyruvate; ROS, reactive oxygen species; SAH, subarachnoid hemorrhage; TCA, tricarboxylic acid cycle; TCD, transcranial Doppler ultrasound flow velocities; VSP, vasospasm

To our knowledge, this is the first study investigating cerebral NO concentrations over a period of 14 days in relation to cerebral metabolism and oxygenation. Pathophysiological considerations of NO mechanisms after SAH in conjunction with our results are shown in Fig. 3.

We observed that the NOx levels have pronounced biphasic kinetics. NOx concentration was high in acute phase of SAH (first 7 days) and decreased substantially in the subacute period (day 8–14). In animal models, a NO decrease was observed within minutes after SAH due to blood extravasation resulting in NO scavenging [40]. This may lead to reduced cerebral blood flow by vasoconstriction and cause platelet aggregation with microthrombosis [38, 40]. This very early phase of SAH is not accessible in SAH patients. However, within 24 h after SAH, NO exceeded baseline values and remained elevated thereafter [51]. In humans, NO in CSF peaked within the first 5 days after SAH, which is consistent with our data from brain parenchyma, where it seems even more pronounced [25]. At the similar time point, day 5 of SAH, Suzuki et al. showed that nitrite and nitrate levels in CSF reached their maximal values, which were five times higher than the healthy controls and decreased gradually thereafter [43]. Furthermore, NOx CSF levels seem to be significantly higher in patients suffering cerebral vasospasm [48]. Overall, concentrations of NOx metabolites in CSF represent approximately 30% of the levels found in cerebral microdialysate [42]. In a microdialysis study measuring NOx in seven patients during the first week after SAH, significantly lower NO levels were observed [34]. Following an initial NOx peak of 46.2 ± 34.8 μM at day 1–3 after SAH, patients showed an exponential decline to 23.5 ± 9.0 μM on day 7. Compared with our patients, NOx peak levels were significantly lower and the decline significantly faster. This might be explained by the higher number of poor-grade patients in our cohort, as previous studies have shown that NOx increase is dependent on the initial clinical status and functional outcome following SAH [42, 43, 48]. Interestingly, NOx concentrations in cerebral microdialysate of electively clipped patients (36.7 ± 9.6 μM; considered as physiological levels) were as low as NO concentrations 2 weeks after SAH in our cohort [35]. These data clearly show that pathologically high concentrations of NO occur mostly during the acute phase following SAH.

The initial increase in NO levels during the acute phase might be explained as a reaction to both neuroinflammation and impaired cerebral perfusion. It has been suggested that SAH induces neuroinflammation due to the release of damage-associated molecular patterns [5]. A microdialysis study has shown a high pro-inflammatory response following SAH with interleukin-6 and matrix metalloproteinase-9 release [13]. Several inflammatory pathways such as nuclear factor “kappa-light-chain-enhancer” of activated B cells are induced and lead to upregulation of iNOS and excessive NO [19, 24]. Cerebral ischemia can also upregulate iNOS via HIF-mediated pathway [14]. In previous animal experiments, SAH was associated with a marked increase and upregulation of iNOS from day 1–7 [32, 36]. The latter is in a good agreement with our data.

We observed that NOx increase was highly associated with cerebral hypoxia, perhaps acting as a compensatory mechanism to improve cerebral perfusion, which is in line with the observed lower TCD flow velocities at elevated NOx levels. Khaldi et al. analyzed relative cerebral NO and ptiO_2_ changes over time and found a positive correlation within the first week after SAH [17]. These concomitant changes support the assumption that NO may increase oxygen delivery by its vasodilatory effect. However, nitrite levels were also found to be up to 8 times higher in microdialysate upon ischemia (80 μM) compared with non-ischemic conditions (10–20 μM) [42]. An increase of nitrite preceded even the development of cerebral infarction by 1–2 days and was significant higher in poor-grade patients.

NO as a potent vasodilator is important in cerebral autoregulation by regulating the vascular tone via eNOS and interaction with soluble guanylate cyclase [10]. In presence of hypoperfusion due to cerebral vasospasm, elevated NO was previously observed in the CSF [48]. The NO formed by eNOS is predominantly associated with the vasotropic effects. However, in patients suffering radiographic vasospasm nitrite, an alternative source of NO was significantly lower compared with patients without cerebral vasospasm [16]. Administration of nitrite may prevent the occurrence of vasospasm [6, 29, 30]. Therefore, these results suggest that NO depletion, e.g., due to decreased eNOS activity, may provoke cerebral vasospasm leading to secondary ischemia [21]. Interestingly, NO depletion in our cohort was observed at the beginning of the subacute period, suggesting a reduction of its vasodilative effect, thereby promoting cerebral vasospasm.

Furthermore, various vasoactive substances released from lysed erythrocytes exert vasotropic effects. Oxyhemoglobin induces transcription and synthesis of ET-1, a potent long-lasting endothelium-derived vasoconstrictor, which increases in plasma within minutes after SAH [20]. Whereas the endothelin-A receptor on smooth muscle cells is mediating vasoconstriction, the endothelin-B receptor on endothelial cells induces vasodilation via eNOS and prostacyclin release [46]. However, under pathological conditions, a decrease in NOS substrates causing uncoupling of eNOS can result in formation of peroxynitrite instead of NO, thereby activating oxidative stress and aggravating vasospasm [32]. Further it has been shown that not ET-1 but asymmetric dimethyl-L-arginine, an endogenous inhibitor of NOS, correlates with the occurrence of cerebral vasospasm, thereby supporting the hypothesis that NO depletion plays a major role in the pathophysiological process of vasoconstriction [15]. We also observed a strong negative correlation of NO with ptiO_2_ and TCD flow velocities, which was more pronounced during the acute phase of SAH. This fact supports our assumption that NO facilitates vasodilation and oxygen delivery, as this has been shown, for instance, in healthy volunteers who manifested hypoxia-induced cerebral vasodilatation mediated by NO [45].

While cerebral hypoxia was only associated with increased lactate and LPR values, increased NOx was associated with accumulation of glycerol, lactate, and pyruvate. In addition to pyruvate, the mean values of another mitochondrial substrate, glutamate were also increased at elevated NOx, but extremely high variation made these changes insignificant. An increase in the levels of these mitochondrial substrates may be indicative of decreased uptake by mitochondria due to mitochondrial dysfunction, suggesting impaired energy metabolism in the brain tissue at high levels of NOx.

The assumed interactions between NO and mitochondria are depicted in Fig. 2e and Fig. 3. The initial excessive increase in NO levels causes formation of reactive oxygen species, such as peroxynitrite, which can damage mitochondria and lead to endothelial dysfunction and apoptosis of smooth muscle cells [39]. Excessively elevated NO may irreversibly inhibit complex I and reversibly inhibit complex IV in mitochondria, thereby causing mitochondrial dysfunction [2]. We observed in our ex vivo experiment that the increment in NO concentration observed in microdialysates during the acute phase inhibits mitochondrial respiration irrespectively whether substrate of complex I or complex II were provided, suggesting that NO predominantly reacts with complex IV. This assumption was supported in this study by direct determination of activity of complex IV, which was decreased in the presence of NO. This is in line with previously published results [3]. Ex vivo addition of myxothiazol to the NO samples showed no effect, confirming that the electron transport chain was already almost fully inhibited by NO. Thus, the amount of NO available in the cerebral interstitial fluid is sufficient to disrupt mitochondrial function, resulting in anaerobe glycolysis instead of aerobic oxidative phosphorylation. Clinically, a strong positive correlation of NOx with cerebral lactate, pyruvate, and glutamate was observed during the acute phase in our cohort and was not evident anymore after NO depletion during the second week. Similar correlations within the first week after SAH were reported by Sakowitz et al., although not as pronounced as in our cohort [34]. In contrast, Carpenter et al. showed an inverse correlation of NO to metabolic parameters of brain injured patients (11 TBI patients and 1 SAH patient) [4]. Early after injury, NO levels were associated with higher cerebral glucose and lower cerebral lactate and LPR values, suggesting increased blood supply and a shift towards improved metabolism. Interestingly, in this cohort, mean NO levels were as low as 32.7 ± 16.8 μM, which represent similar NO values as observed in microdialysate of electively clipped patients [35]. In our population, NO levels were initially 3 times higher and reached these levels not before day 13–14 after SAH, which were considered as the return to physiological background levels. This discrepancy of improved metabolism at lower NO levels and the accumulation of mitochondrial substrate at higher levels supports our hypothesis that elevated NO levels critically influence energy metabolism in brain tissue affected by SAH.

Several limitations of this study deserve mentioning. As this was an exploratory study, the results must be interpreted with caution and conclusions are limited to generation of new hypotheses. Despite the large SAH population referred to our institution, the number of patients requiring invasive multimodality neuromonitoring is limited. Therefore, the number of patients included in this study was small. Furthermore, monitoring of NO and cerebral metabolites using cerebral microdialysis is a focal measurement and thereby limited to a small parenchymal area. This limitation was met by placement of the probe ipsilaterally to the ruptured aneurysm and/or maximal extension of subarachnoid blood clot to monitor the area with the highest likelihood developing delayed cerebral ischemia. As NO concentrations and multimodality monitoring values were averaged for each day, transient changes were not registered.

In conclusion, cerebral NO concentrations are highly elevated within the first 7 days after SAH, predominantly from inflammatory sources based on the literature [14, 37]. In vivo elevated NO levels were strongly correlated with cerebral lactate and pyruvate levels (and LPR), as well as with the accumulation of mitochondrial substrate, i.e., glutamate, thereby indicating mitochondrial dysfunction and anaerobic shift in metabolism. Activation of anaerobic metabolism often termed tissue hypoxia can be due to either impaired oxygen delivery (ischemia) or impaired cellular oxygen uptake/utilization, such as inhibition of mitochondrial function or a combination of both factors. We do not know exactly the contribution of impaired oxygen delivery and of inhibition of mitochondria to the activation of anaerobic metabolism, but our ex vivo data suggest that the amount of NO formed in the lesion area alone can be sufficient to completely inhibit mitochondria and switch to anaerobic metabolism. Further studies are required to understand mechanisms underlying this observation.
